# Use of and Beliefs toward Novel Tobacco and Nicotine Products among Portuguese University Students: A Pandemic Survey

**DOI:** 10.3390/ijerph21040478

**Published:** 2024-04-14

**Authors:** Marina Vaz, Pedro Cascais, Olga Lourenço

**Affiliations:** 1 FCS-UBI, Faculty of Health Sciences, University of Beira Interior, Avenida Infante D. Henrique, 6200-506 Covilhã, Portugal; marina.vaz@ubi.pt (M.V.); pedro.cascais@ubi.pt (P.C.); 2 CICS-UBI, Health Sciences Research Centre, University of Beira Interior, Avenida Infante D. Henrique, 6200-506 Covilhã, Portugal

**Keywords:** electronic cigarette, heated tobacco, nicotine, tobacco, university students, water pipe

## Abstract

Over the last decade, novel tobacco and nicotine product experimentation and use have dramatically increased among the youth, even in countries with strong tobacco control and anti-smoking social norms. We performed an online questionnaire-based cross-sectional study in March-June 2021, targeting students from the University of Beira Interior, Portugal. The aim was to assess the experimentation and use of tobacco and nicotine products and students’ beliefs towards these products. Of the 452 participants, 67.0% were female; the mean age was 21.9 ± 3 years. Most students (60.4%) reported experimenting with tobacco/nicotine products; 31.2% were current users; polyconsumption was common. Of the current users, all used cigarettes, 41.1% used heated tobacco, 20.6% e-cigarettes, and 14.9% used water pipes. Our multivariate analysis showed that being male, being in the third year of study, and cohabiting or socializing with smokers were strongly associated with tobacco/nicotine use. While most students agree that heated tobacco and e-cigarettes are addictive, that they are not less harmful than cigarettes, and that second-hand exposure may cause health problems, few tobacco/nicotine users are ready to quit, and few students support a smoke-free university campus. These findings indicate high experimentation and the regular dual use of novel tobacco and nicotine products and suggest a pro-smoking social norm among university students.

## 1. Introduction

Tobacco consumption stands as a pressing global public health issue. According to the World Health Organization (WHO), tobacco claims the lives of approximately half of its users annually, resulting in over 8.2 million deaths, with 1.2 million attributed to exposure to second-hand smoke [1]. In Portugal, smoking is the leading cause of loss of healthy life years [2]. Recent data from the Institute for Health Metrics and Evaluation (IHME) indicate that tobacco-related diseases led to over 13,500 deaths in Portugal in 2019 [3]. While the prevalence of daily smokers in Continental Portugal is on a decline, currently at 14.2% among individuals aged 15 or older [4], nearly half of all 18-year-olds have experimented with tobacco, with 40% reporting regular usage, making tobacco the second most consumed substance in this age group after alcohol [5]. In Europe, the prevalence of young smokers is estimated to be 20%. Despite the downward trend in traditional tobacco consumption, the use of emerging tobacco and nicotine products is on the rise [6].

University students are particularly vulnerable to initiating psychoactive substance use, including tobacco, as university represents a transitional phase where social identities are developed and individuals face new challenges and peer pressure [7]. Moreover, many young individuals underestimate the risks associated with tobacco use and perceive themselves as invulnerable to its harms [8]. This phase often marks the transition from experimentation to regular tobacco use, with young adults developing nicotine dependence. One study by Ravara et al. (2014) has shown that half of smoking physicians begin regular smoking during their university years, a trend persisting for over three decades [9,10]. In Portugal, the tobacco industry has launched various promotional initiatives targeting young people, such as expanding sales outlets at music festivals, online sales, and social media promotions through using influencers [2].

Electronic cigarettes, also known as e-cigarettes, are electronic devices capable of generating nicotine-containing aerosols by heating a prepared solution [11]. Invented in China in 2003, they gained popularity in Europe by 2006, particularly among young people and adolescents [12,13]. According to data from the 2020 Special Eurobarometer 506 [14], e-cigarette use in Europe is estimated to have a 2% prevalence, with younger respondents showing a higher likelihood of experimentation. In Portugal, e-cigarettes rank as the second most consumed nicotine product after conventional tobacco, with 1.6% of the population aged 15 and over reporting daily or occasional use [2,4]. Additionally, it is important to note the accessibility of a wide range of e-cigarette products, including disposable devices, those infused with nicotine salts, devices featuring disposable pre-filled cartridges, and refillable options compatible with e-liquids, facilitated by their widespread availability on the internet [15,16]. Highlighting the inherent risks associated with these products, including the potential for content modification to increase nicotine levels or incorporate other substances, is crucial. Such accessibility presents significant challenges and emphasizes the necessity for a comprehensive understanding of these emerging products, as well as the need to regulate them [17].

Heated tobacco products represent another emerging category; they allow for the heating tobacco without combustion to produce an aerosol devoid of the characteristic odor of burnt tobacco [18]. The Conference of the Parties (COP) of the WHO Framework Convention on Tobacco Control (WHO FCTC) held in Panama City in 2023 classified aerosols of novel and emerging tobacco products as “tobacco smoke” [19]. In 2020, the prevalence of heated tobacco consumption in Europe was 1%, with the younger population being the main consumers [20]. These products are on the rise, particularly among young people with higher socioeconomic status [20]. However, due to their novelty, the long-term health effects of electronic cigarettes and heated tobacco remain poorly documented, with many studies being carried out amidst significant conflicts of interest, as they are often conducted or funded by producers of these products.

The water pipe, also known as shisha or narghile, has gained popularity in recent years, especially among young people [21]. Data from the Special Eurobarometer 506 indicates a rise in water pipe experimentation, with 29% of individuals aged 15 to 24 having tried it, and 6% reporting monthly consumption [21]. This trend is fueled by misconceptions about its health effects and its association with socializing among young people [22,23]. As with other emerging tobacco products, aerosols of the water pipe tobacco are also classified as “tobacco smoke” by the WHO FCTC [19]. However, water pipe smoking exposes individuals to significantly higher concentrations of toxic substances compared to conventional cigarettes, raising serious health concerns [24]. Additionally, most users of these new tobacco products consume them alongside traditional cigarettes, exacerbating their harmful effects [25]

In addition to the public health implications, smoking imposes financial burdens on both consumers and governments, with direct expenses for acquisition and increased healthcare costs, as well as social and environmental consequences [2]. Recognizing smoking as a priority health issue, Portugal has implemented measures such as the National Tobacco Control Program (PNPCT), based on the WHO Framework Convention on Tobacco Control and the MPOWER strategies [26,27,28]. Recent legislative amendments have extended smoking bans to include new tobacco products, reflecting ongoing efforts to address emerging challenges [29,30].

Despite these initiatives, there is limited research on tobacco consumption and control measures among university students, and the effectiveness of prevention policies in this specific context is unknown. This study aimed to evaluate rates regarding experimentation with and the consumption of tobacco and nicotine products among students at the University of Beira Interior (UBI) and the factors associated with their use.

## 2. Materials and Methods

### 2.1. Study Design and Population

A cross-sectional observational research study was carried out through an online survey between 8 March and 12 June 2021 at UBI. Participants were recruited via an email from the Public Relations Office, by sharing study details on social networks such as Facebook and Instagram, and word of mouth. All students enrolled at UBI in the academic year 2020/2021 (a total of 8223 students) from any of its 5 faculties were eligible to be included, and our study was based on a convenience sample. Participation in the study was voluntary and anonymous, and participants had the right to refuse to participate without giving a reason. The study proposal was reviewed and approved by the UBI Ethics Committee, Covilhã, Portugal (process number: CE-UBI-Pj-2020-089:ID394).

### 2.2. Questionnaire and Study Measures

The research tool was a questionnaire (Appendix A Table A1) developed using standardized international tools validated in previous studies, such as the Fargeström test [31] and the smoker’s motivation stage [32]. Questions related to students’ opinions about tobacco and nicotine products (e-cigarettes, heated tobacco, and water pipes) and about the regulation of exposure to tobacco smoke were developed based on previous studies [26,33]. The questionnaire, divided into 6 sections, included 62 questions addressing personal characteristics and related to attitudes and behaviors towards various tobacco and nicotine products. For the preparation of the final version of the questionnaire, a pilot study was carried out on a group of students who evaluated the understanding and organization of the questions, their difficulty in answering them, and the duration needed to complete the questionnaire.

Section I included questions on sociodemographic characteristics: age, gender, faculty, course, course year, number of enrolments, place of residence (during and outside the school term), and exposure to tobacco smoke (at home, at the faculty, and in leisure or social spaces).

Section II included questions on experimentation with and consumption of tobacco (conventional cigarettes, cigarillo, hand-rolled cigarettes, heated tobacco, water pipes, tobacco with other substances or drugs) and nicotine products (e-cigarettes).

Section III was based on the Fagerström test [31], a standard instrument for assessing the intensity of physical addiction to nicotine. In scoring the Fagerström test, yes/no items were scored either 0 or 1, and multiple-choice items were scored from 0 to 3. The items were summed to yield a total score of 0–10. The higher the total Fagerström score, the more intense the physical nicotine dependence. Smokers who scored 7 or more points were considered “highly dependent”, and those with a score equal to or less than 3 were considered to be “low dependent”. For this study, “moderate dependence” was considered for scores between 4 and 6.

Section IV included questions on experimentation with and frequency of consumption of heated tobacco, water pipes, and e-cigarettes.

Section V inquired about the opinion and/or knowledge of students about the health effects and harm perception of tobacco and nicotine products. Responses to these questions were categorized using a 5-point Likert scale (“strongly disagree”, “disagree”, “neither disagree nor agree”, “agree”, and “strongly agree”).

Section VI inquired about the students’ opinions on the current smoking ban and its compliance in the faculty and hospitals/health services, in addition to their thoughts on a broader ban on UBI and the idea of a “Smoke-free campus”.

Within the scope of this study, the following operational definitions were applied:

- Smoker/user (regular user of tobacco and/or nicotine products)—Respondents who have smoked at least 100 cigarettes (or a similar amount of other tobacco or nicotine products) in their lifetime and who reported tobacco or nicotine product use in the last 30 days;
- Non-smokers/non-users (non-users of tobacco and/or nicotine products)—Respondents who reported having smoked fewer than 100 cigarettes (or a similar amount of other tobacco products) during their lifetime and/or do not currently smoke/use tobacco and/or nicotine products;
- Daily user of tobacco and/or nicotine products–Respondent who answered, “I smoke/use daily” to the question “How often do you smoke/use an e-cigarette?”
- Occasional user of tobacco and/or nicotine products—Respondents who answered, “I smoked/used in the last 30 days” or “I smoked/used in the last week” to the question “How often do you smoke/use an e-cigarette?”

We also asked the respondents about the use of e-cigarettes using the question “ Have you ever used or use e-cigarettes?” We considered e-cigarette or vape consumers as those respondents who consume e-cigarettes “Once a month”, “Once a week”, or “Once a day”.

In addition to these sections, the questionnaire also contained information regarding the study, its objectives, the Ethics Committee approval process number, the type of response (anonymous, confidential, and voluntary), the possibility of withdrawing at any time during participation in the study, and the contact details for the research team members.

In the first question of the questionnaire, the participant declares if they accept to participate in the study after reading information about it, thus allowing them to essentially sign an Informed Consent Statement.

### 2.3. Statistical Analysis

For statistical analysis, Microsoft Office Excel version 2023 and IBM SPSS Statistics 28 (IBM, Armonk, NY, USA) were used. Categorical variables were described through their respective absolute and relative frequencies (percentages). Quantitative (continuous) variables were described using measures of central tendency and dispersion (mean, standard deviation, and variance).

A univariate and bivariate descriptive analysis of the variables under study was performed. To verify a possible relationship between these variables, Pearson’s Chi-Square test was used with a statistical significance level of 5% (*p* < 0.05). When appropriate, the value of the Odds Ratio (OR) and Confidence Intervals (CIs) of 95% (*p* < 0.05) are presented.

To assess the association between independent and dependent variables, a multivariate analysis was performed, eliminating confounding through binary logistic regression. The dependent variable was used in its dichotomous form (non-user, user), while the original categories were kept for the independent variables, except for the variable “Year of attendance/Study cycle”, whose categories were recoded to “1st”, “2nd”, and “3rd year of the cycle degrees” and “master, postgraduate and doctoral degree”. The “Forward” process was used for the selection of variables; this type of algorithm starts from a model without predictors, to which the independent variables are added sequentially, thus arriving at an optimized solution with several independent variables. The results of this analysis are presented as regression coefficients, ORs, or CIs and were validated through the use of the Hosmer and Lemeshow test (*p* < 0.05).

The variable “age”, which assumed the values of 18, 19, 20, 21, 22, 23, 24, 25, 26, 27, 28, 29, 30, and “Over 30 years”, was recoded by defining intervals (age groups): “18–22”, “23–26”, “27–30”, and “30+”.

The variable “Course” was recoded in a dichotomous way, reflecting the field of study of each respondent as follows: “Health”—Ph.D. in Biomedicine, Ph.D. in Biochemistry, Ph.D. in Pharmaceutical Sciences, degree in Biochemistry, degree in Biomedical Sciences, degree in Optometry and Vision Sciences, master in Optometry and Vision Sciences, integrated master in Pharmaceutical Sciences, integrated master in Medicine, and postgraduate course in Telehealth; “Other”—all other courses.

The “Year” variable was recoded to reflect the study cycles of the respondents: “1st”, “2nd”, and “3rd” year corresponded to respondents in the 1st, 2nd, or 3rd year, respectively, of a first-cycle degree or an integrated master’s degree; “Master’s and Postgraduate” included respondents in the 4th, 5th, or 6th year of integrated master’s degrees, as well as those pursuing any second-cycle degrees; “Ph.D.” includes respondents who were pursuing any third-cycle degree (regardless of the year).

The 5-point Likert scale used in sections V and VI of the questionnaire was dichotomously recoded into “agree” (“agree”, “totally agree”) and “disagree” (“completely disagree”, “disagree”, “neither disagree nor agree”).

## 3. Results

### 3.1. Characteristics of the Study Population

Completed questionnaires were obtained from 455 individuals, with an overall response rate of 5.5%. This analysis was based on the responses to the questionnaire received from 452 students (67.0% females) with a mean age of 21.9 years. The distributions of gender, age, faculty, study field, and year are presented in Table 1.

### 3.2. Experimentation with Traditional Cigarettes, Heated Tobacco, E-Cigarettes, and Water Pipes

**Figure 1:**
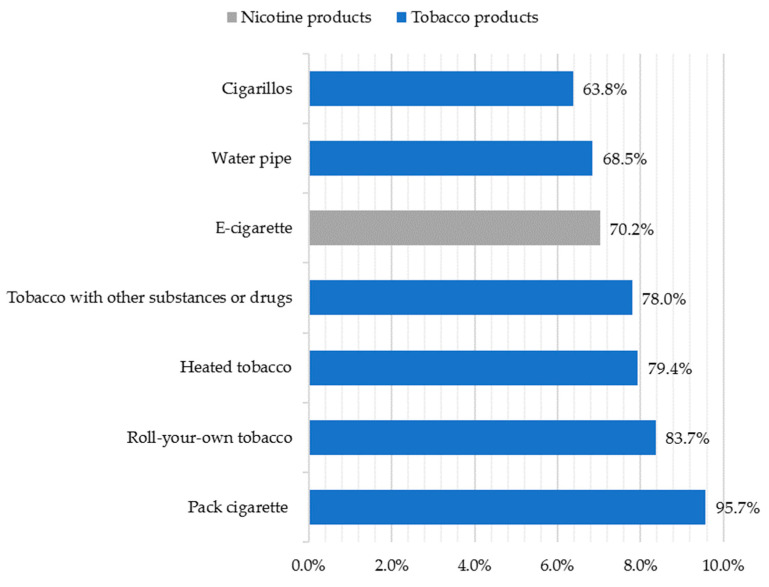
Tobacco and nicotine products experimented with among tobacco and/or nicotine users (*n* = 141).

The majority of respondents (60.4% (*n* = 273)) had experimented with tobacco and/or nicotine products; however, most students were non-users (68.8%), 20.1% were daily users, and 11.1% were occasional users. Considering those who use regularly (*n* = 141), all had experimented with at least one tobacco product, but only 70.2% (*n* = 99) had experimented with nicotine products (Figure 1).

### 3.3. Regular Use of Traditional Cigarettes, Heated Tobacco, E-Cigarettes, and Water Pipes

Boxed cigarette use was reported by 73.8% of users, heated tobacco use by 41.1%, and roll-your-own cigarette use by 32.6%, while e-cigarettes were used by 20.6% of students.

**Figure 2:**
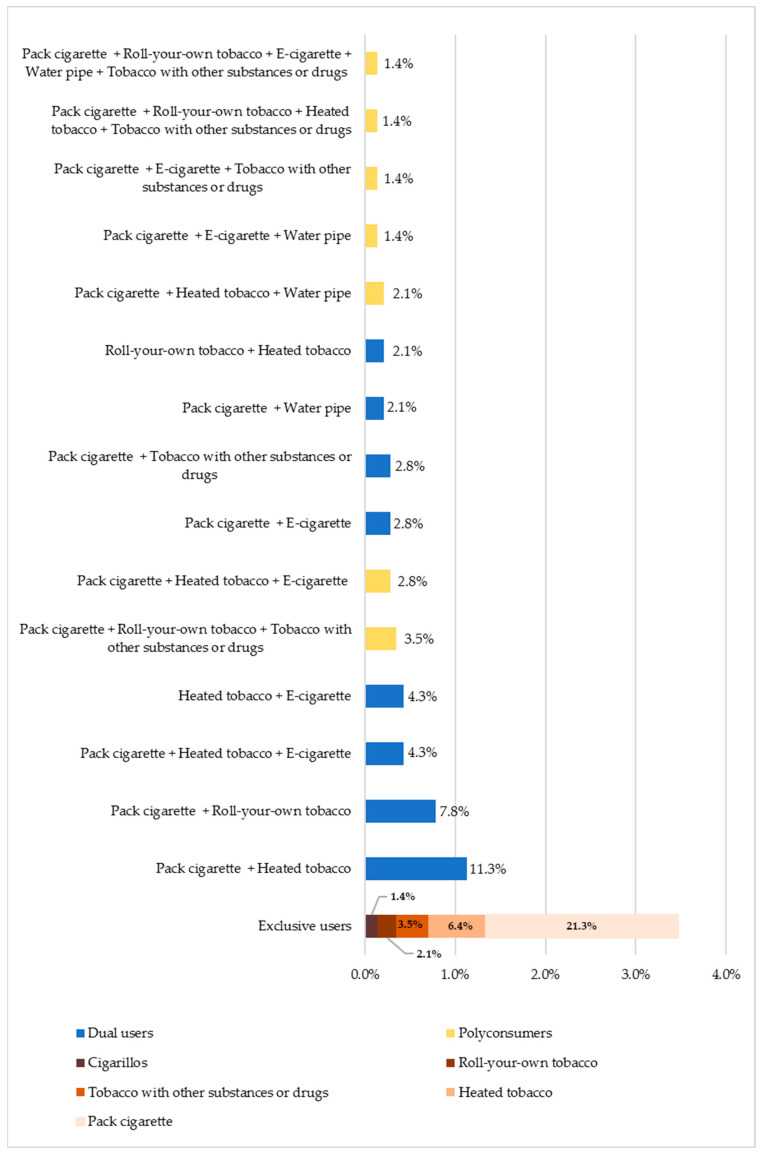
Consumption patterns of tobacco and nicotine products (*n* = 141).

About one third (34.8%) of tobacco and/or nicotine users use only a single product, namely boxed cigarettes (21.3%), heated tobacco (6.4%), tobacco mixed with other substances/drugs (3.5%), hand-rolled cigarettes (2.1%), or cigarillos (1.4%). Concerning dual consumers, these individuals predominantly use boxed cigarettes and heated tobacco simultaneously (11.3%, corresponding to 31.4% of dual consumers) and boxed cigarettes with roll-your-own cigarettes (7.8%, corresponding to 21.6% of dual consumers). Polyconsumers account for 29.1% of the surveyed users, with “boxed cigarettes, heated tobacco, and electronic cigarettes” (4.3%, corresponding to 14.6% of polyconsumers) and “boxed cigarettes + roll-your-own tobacco + tobacco mixed with other substances or drugs” (3.5%, corresponding to 12.2% of polyconsumers) as the main combinations (Figure 2).

**Figure 3:**
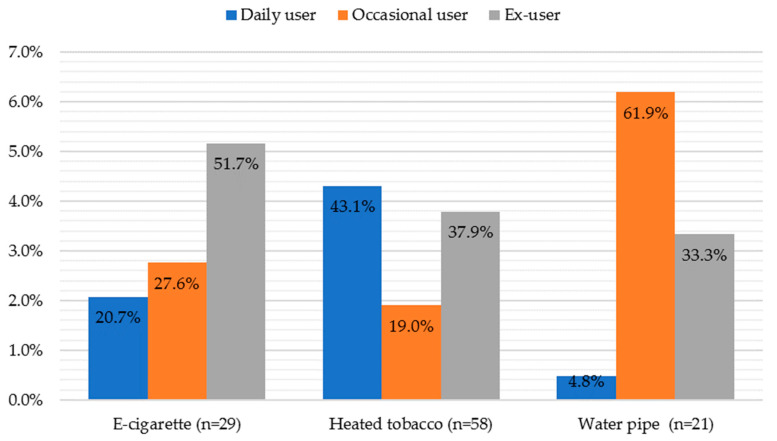
Consumption patterns of products that are alternatives to traditional cigarettes.

The patterns of consumption of products that are alternatives to traditional cigarettes are very different (Figure 3). The e-cigarette is, of the three products, the one whose percentage of former users was higher (51.7%). Heated tobacco was the product found to be consumed daily the most (43.1%), the product that is the least likely to be consumed occasionally (19.0%), and the one with the lowest number of former users (9%). The most common pattern of water pipe use was occasional, with only one user reporting daily use.

Smoking status differed according to sex and year of study. The male students showed more frequent tobacco and/or nicotine product use compared to the female students (45.6% vs. 24.4%). Students in the third year of a first-cycle degree showed the highest use of tobacco and/or nicotine products (45.1%). In contrast, the use of tobacco and/or nicotine products of the Ph.D. students was the lowest (8.3%). It was also possible to verify an increase in use throughout the first cycle. Details are presented in Table 2.

The use of tobacco and/or nicotine products by respondents who live, study, or socialize with smokers is significantly higher than that of those who do not (Table 3).

### 3.4. Association between Sociodemographic Characteristics and Proximity to Smokers/Users and Use of Tobacco and/or Nicotine Products

To eliminate confounding, logistic regression was performed to confirm the existence of an association between the use of tobacco and/or nicotine products and the variables that showed a significant relationship with it in the bivariate analysis (gender, year, living/socializing with smokers/users) (Table 4).

Respondents who preferred not to reveal their sex and those who did not answer the question “Do any of the people you live with during school term smoke/use tobacco or nicotine products?” were not included.

The logistic regression model was significant (Chi-Square= 76.486, *p* < 0.001), explained 26.4% (R^2^_Nagelkerke) of the variations in tobacco and/or nicotine use, and correctly classified 72.2% of cases, and there were no significant differences between the classifications performed by the model and the observed reality (Hosmer and Lemeshow test: *p* = 0.860).

The possibility of being a user is three times higher for male students; two times higher for students in the third year of a first-cycle degree; three times higher for students who live with smokers/users of tobacco or nicotine products during the school term; two times higher for students whose household includes smokers/users of tobacco or nicotine products; and six times higher for students who socialize with smokers/users of tobacco or nicotine products within the faculty. In the binary logistic regression model, there was no influence (*p* > 0.05) of age, faculty, or study field on the possibility of being a user.

### 3.5. Physical Dependence on Nicotine, the COVID-19 Pandemic, and Smoking Cessation

According to the results of the Fagerström test, most smokers (76.6%) had a low nicotine dependence, 18.4% had a moderate nicotine dependence, and 5.0% had a low dependence.

The COVID-19 pandemic influenced the use of tobacco products and/or nicotine in 75.2% of the users. More than a quarter of respondents (28.4%) reported an increase in use, 40.4% reported a decrease, and 6.4% stopped smoking/using. Nevertheless, when asked about their intentions to quit smoking, most respondents (55.3%) revealed that they wanted to do so but not within the next 6 months. Of the remaining students, 18.4% did not intend to quit smoking, 13.5% wanted to do so in the next 6 months, and only 12.8% intended to quit smoking in the next month (Table 5).

### 3.6. Opinions and Beliefs on E-Cigarettes, Heated Tobacco, and Water Pipes among Students

Most respondents agree that exposure to the aerosol from e-cigarettes can be harmful to health, that e-cigarettes are addictive, and that advertising/marketing increases the number of users. Furthermore, most consider that the use of e-cigarettes should be prohibited in closed spaces. On the contrary, respondents do not agree that e-cigarettes are less harmful to health than traditional cigarettes or that they help in smoking cessation (Table 6). Moreover, considering the Odds Ratio analysis, the possibility of a user agreeing with the statement that electronic cigarettes are less harmful to their users than conventional cigarettes is (approximately) three times higher than the possibility of a non-user agreeing with this statement.

A minority of respondents (23.2%) agree that heated tobacco is less harmful to users’ health than traditional cigarettes and that it helps in smoking cessation. A larger fraction (41.4%) believe that it fosters an idea of integration and social acceptance, or even superiority, among users. On the other hand, the overwhelming majority of these respondents argue that heated tobacco is harmful due to the risk of exposure to its aerosol, which creates dependence; that its consumption should be prohibited in enclosed spaces; and that advertising the product increases the number of users (Table 7). Contrary to the results found among non-users of heated tobacco, most users believe that heated tobacco is less harmful to health than traditional cigarettes and do not agree with the ban on its use indoors.

Most respondents (63.1%) agree that the water pipe is becoming increasingly popular among young people, but only 7.7% believe that it helps in smoking cessation. The general agreement rate for the remaining statements varied between 30 and 40%. There were no significant differences in the responses given between those who have used the water pipe and those who did not (Table 8).

## 4. Discussion

The present study assessed tobacco and nicotine product experimentation and consumption among UBI students, identifying the factors associated with this consumption and the students’ opinions on emerging nicotine and tobacco products. It also evaluated students’ opinions on compliance with the law prohibiting smoking in enclosed public places and their agreement regarding a potential extension of regulations to outdoor university spaces. A better understanding of tobacco and nicotine consumption among university students could contribute to improving the control of this epidemic by implementing strategies specifically targeted to the studied population.

The majority of respondents had already experimented with tobacco and/or nicotine products, and 3 out of 10 of these students consumed these products regularly; all had consumed tobacco products (31.2% of respondents), and only 6.4% had consumed nicotine products (e-cigarettes). The prevalence of tobacco product consumption found in this study aligns with that of studies conducted on university students in Germany (2017) [27], Poland, and Italy (2020) [28]; however, it is relatively higher than that national studies, namely that of Esteves et al., conducted at UBI in 2017 [34], and Alves et al., conducted at the University of Minho in 2020 [29]. In both studies, only 1 in 10 respondents was considered a smoker.

The average age at which respondents began experimenting with tobacco and/or nicotine products was 15.5 ± 2, consistent with the literature [14,29]. Adolescence is characterized by being a phase of life marked by changes at the physical, mental, and social levels, making this population more susceptible to risky behaviors such as tobacco and/or nicotine product experimentation and/or consumption [35].

Most respondents reported having experimented with and regularly consuming more than one tobacco and/or nicotine product, which is consistent with previous studies, notably that of Butler et al., which was conducted at an American university in 2015. Polyconsumption is popular among young people, although it may entail a greater health risk than the consumption of only one product and may enhance dependency [30,36].

Approximately 6% of respondents reported consuming electronic cigarettes, of which 2 out of 10 consume them daily. The prevalence of electronic cigarette consumption is significantly lower than that reported by other studies, particularly that of Jones et al. (conducted on American university students aged 18 to 25 in 2019 and 2020) [37]. On the other hand, Daniel et al., in 2021, reported a daily consumption pattern in American university students similar to that of this study [38].

Regarding heated tobacco, about 13% of respondents reported consuming this product, of which 4 out of 10 consume it daily. The prevalence of heated tobacco consumption is, like that for electronic cigarettes, higher than that found in other studies, particularly that of Majek et al., conducted in 2019 among medical students in Poland [39]. Majek et al. also compared the use of traditional cigarettes, heated tobacco, and electronic cigarettes, and similar to this study, traditional cigarettes were the most consumed product. However, electronic cigarettes proved to be more popular than heated tobacco, in contrast to this study.

The water pipe is used by university students, mostly socially, in cafes and bars and is associated with socializing and fun, justifying the occasional consumption pattern observed in this study [23]. The prevalence of water pipe consumption was approximately 5%, a value lower than that deduced by Fevrier et al. in 2018 in a study conducted on American university students [40].

Male students reported higher consumption of tobacco and/or nicotine products compared to female students, in line with the study by Lavado et al., which was conducted in 2019 and focused on Portuguese 18-year-olds [5]. On the other hand, when comparing the consumption reported by respondents pursuing bachelor’s degrees, a progressive increase was observed, with third-year respondents reporting higher consumption than those in other years, which was also observed in a study conducted by Alves et al. at the University of Minho in 2019 [29]. Thus, this trend suggests that the academic environment promotes the consumption of tobacco and/or nicotine products.

It was also found that living and socializing with other smokers influences the consumption of tobacco and/or nicotine products. The influence of peers on the consumption of such products, mainly tobacco, by young people is well documented and aligns with the results of this study. Windle et al. (2017) define that the consumption of substances such as tobacco, alcohol, and cannabis by parents, siblings, and friends is related to the use of these products by university students [41].

Eight out of ten consumers of tobacco and/or nicotine products presented low nicotine dependence according to the Fagerström test. However, despite the majority intending to quit smoking, only one in ten stated that they intended to do so in the next month. These results are consistent with the observations made by Soares et al. in a study conducted on UBI medical students in 2016 [33], highlighting the need to raise awareness among students about the importance of ceasing their consumption of tobacco and/or nicotine products.

More than three-quarters of tobacco and/or nicotine product consumers changed their consumption patterns during the COVID-19 pandemic. Although more than a quarter of tobacco and/or nicotine product consumers increased their consumption, most reduced their use of them or ceased using them. Movement restrictions, the possibility of some respondents returning to their family home, difficulty accessing to tobacco products, and the uncertainty of the influence of smoking on the effects of the disease may justify this decrease [42].

It was found that most respondents do not consider new products to be less harmful to health than traditional cigarettes. However, being a consumer of electronic cigarettes and heated tobacco is a predictive factor for not agreeing with such a statement regarding the consumed product. According to the National Program for Tobacco Prevention and Control report of 2020, believing that alternative products to common cigarettes are less dangerous to health than smoking tobacco is a reason for consuming electronic cigarettes and heated tobacco cited by a large portion of Portuguese consumers [2].

Most respondents believe that exposure to the environmental aerosol from electronic cigarettes and heated tobacco is harmful to health but do not consider exposure to water pipe aerosol harmful. In addition, of the three products, the respondents only consider the water pipe not to be addictive. The lack of knowledge about the addictive and harmful properties of water pipes was also shown by Fevrier et al. [40] and Krenik-Matejcek et al. [43] in studies conducted in 2017 and 2018 at American universities.

The prohibition of electronic cigarettes and heated tobacco consumption in enclosed public places is accepted among most respondents. However, heated tobacco consumers believe that this prohibition should not apply to this product. Most respondents believe that the industry’s advertising for electronic cigarettes and heated tobacco increases the number of consumers. Such an opinion alerts us to the need for the regulation and prohibition of advertising for emerging tobacco and nicotine products. Regarding the law prohibiting smoking in enclosed public spaces, approximately 8 out of 10 respondents believe that this is complied with at their university and in health services/hospitals. Most consumers of tobacco and/or nicotine products, unlike non-consumers, do not advocate for this law to be applied throughout the university’s outdoor premises. Finally, most respondents do not believe that the ban on smoking in enclosed public spaces contributes to reducing the number of smokers, in line with the findings of Soares et al. [33,44].

The results of this study raise relevant questions and present worrying aspects, namely (a) the early age at which people begin experimenting with tobacco and/or nicotine products; (b) the high prevalence of consumers of tobacco and/or nicotine products; (c) the nearly widespread polyconsumption; (d) the increase in the consumption of tobacco and/or nicotine products over the course of students’ bachelor’s degrees; (e) the effect that proximity to other smokers has on the consumption of tobacco and/or nicotine products, mainly in the context of socializing within the university; (f) the low number of consumers considering quitting smoking in the next month or in the next 6 months; (g) the high percentage of consumers who believe that their consumption increased during the COVID-19 pandemic; (h) the high percentage of respondents who believe that electronic cigarettes and heated tobacco foster the idea of social integration, or even superiority, among their consumers and that the water pipe is becoming more popular among young people; and (i) the lack of widespread information about the risks and addictive properties of water pipes. In this sense, it is necessary to adopt new measures to address such problems in the university population, such as increasing taxes on all types of tobacco and nicotine products, leading to price increases; conducting campaigns on social media about nicotine and tobacco products to educate about the dangers of these products and combat tobacco industry marketing; creating smoke-free environments, particularly at the university and adjacent locations such as the Academic Bar (prohibition of consumption of any tobacco or nicotine product indoors and outdoors, as well as all activities related to the promotion, sponsorship, and sale of these products); and creating incentives for university students who demonstrate a willingness to quit smoking, such as creating “Quit and Win” contests. Health professionals play a fundamental role in controlling the consumption of these products, particularly in the case of pharmacists, due to their closer proximity to the population. It is essential for pharmacies, especially those located in cities with better education, such as Covilhã, to direct existing smoking cessation measures to the younger population, understand the needs and motivations of this population, and find healthier alternatives.

This study has several limitations that should be considered when analyzing its results. Being an observational cross-sectional study, it does not allow for establishing causality relationships or evaluating the consumption of these products over time, which would be possible with a longitudinal study following students throughout their academic journey. The study relied on self-reported questionnaires, which introduces the possibility of social desirability bias and memory bias, particularly regarding the retrospective evaluation of behaviors associated with the consumption of these types of products, which are often underestimated. Furthermore, some discrepancies were observed in the responses throughout the questionnaire, particularly regarding experimentation with and the consumption of electronic cigarettes, heated tobacco, and water pipes. The study employed a non-random convenience sampling method, which may have led to selection bias. Additionally, the study had a very low response rate, likely influenced by factors such as survey fatigue, spam filters, and competing priorities among potential participants. Thus, the obtained results may not be representative of the study population (UBI students). Furthermore, as the study was conducted at only one university, these results cannot be generalized to the broader Portuguese university-going population.

## 5. Conclusions

The prevalence of tobacco and/or nicotine product experimentation and consumption is high among UBI university students. The predominant patterns are daily consumption and polyconsumption. The most frequently consumed products, in descending order, are traditional cigarettes, heated tobacco, electronic cigarettes, and water pipes. Being male, being in the third year of a bachelor’s degree, and living and socializing in college with other smokers are associated with the consumption of tobacco and/or nicotine products. The majority of students do not support the ban on smoking on the exterior premises of each college, and most users of tobacco and nicotine products do not want to cease their consumption, suggesting a predominantly pro-smoking social norm among university students. The study results strongly suggest that the academic environment promotes the consumption of tobacco and nicotine products. It is urgent to implement robust tobacco prevention and control strategies targeting university students.

## Figures and Tables

**Table 1:** Sociodemographic characterization of the study population (*n* = 452).

Variable		Frequency (*n*)	Percentage (%)
Gender	Male	147	32.5
	Female	303	67.0
	Other	2	0.4
Age (years)	18–22	321	69.0
	23–26	94	20.8
	27–30	25	5.5
	30+	21	4.7
Faculty	Arts and Letters	49	10.8
	Sciences	42	9.3
	Health Sciences	171	37.8
	Human and Social Sciences	128	28.3
	Engineering	62	13.7
Study Field	Health	194	42.9
	Other	258	57.1
Year	1st year	84	18.6
	2nd year	98	21.7
	3rd year	102	22.6
	Master’s and Postgraduate	156	34.5
	PhD	12	2.7

**Table 2:** Bivariate analysis regarding the use of tobacco and/or nicotine products and sociodemographic characteristics.

Variable		Use of Tobacco and/or Nicotine Products		Total *n*	*p*
		Non-User % (*n*)	User % (*n*)		
Sex	Male	54.4 (80)	45.6 (67)	147	**<0.001**
	Female	75.6 (229)	24.4 (74)	303	
Age (years)	18–22	69.9 (218)	30.1 (94)	312	0.136
	23–26	64.9 (61)	35.1 (33)	94	
	27–30	56.0 (14)	44.0 (11)	25	
	30+	85.7 (18)	14.3 (3)	21	
Faculty	Arts and Letters	62.0 (31)	38.0 (19)	50	0.377
	Sciences	77.5 (31)	22.5 (9)	40	
	Health Sciences	72.1 (124)	27.9 (48)	172	
	Human and Social Sciences	65.1 (84)	34.9 (45)	129	
	Engineering	67.2 (41)	32.8 (20)	61	
Study field ^1^	Health	73.2 (142)	26.8 (52)	194	0.083
	Other	65.5 (169)	34.5 (89)	258	
Year	1st year	78.6 (66)	21.4 (18)	84	**0.003**
	2nd year	71.4 (70)	28.6% (28)	98	
	3rd year	54.9 (56)	45.1 (46)	102	
	Master’s and Postgraduate	69.2 (108)	30.8 (48)	156	
	Doctorate	91.7 (11)	8.3 (1)	12	

^1^ a bivariate analysis of use by sex was performed among students in the health study field vs. the other students; this analysis did not show any significant differences (data not shown—Significance = 0.126 (male) and 0.0286 (female). Statistically significant values are marked in bold.

**Table 3:** Bivariate analysis regarding the use of tobacco and/or nicotine products and cohabitation/socialization with users.

Variable		Use of Tobacco and/or Nicotine Products		Total *n*	*p*
		Non-User % (*n*)	User % (*n*)		
Live with users during the school term ^1^	No	83.5 (162)	16.5 (32)	194	**<0.001**
	Yes	56.4 (127)	43.6 (98)	225	
Users in the household	No	75.6 (226)	24.4 (24.4)	299	**<0.001**
	Yes	55.6 (85)	44.4 (68)	153	
Socialize with users at the faculty	No	93.4 (71)	6.6 (5)	76	**<0.001**
	Yes	63.8 (240)	36.2 (136)	376	

^1^ the question “Do any of the people you live with during school term smoke/use tobacco or nicotine products?” was only answered by students who changed residence during the school term. Statistically significant values are marked in bold.

**Table 4:** Binary logistic regression on the use of tobacco and/or nicotine products.

Independent Variable	Regression Coefficient	Wald Test	Sig.	OR	95% CI	
					Min.	Max.
Sex (Male)	0.972	15.630	<0.001	2.643	1.633	4.280
Year (3rd year)	0.798	7.275	0.007	2.222	1.244	3.969
Live with smokers/users during school term (Yes)	1.113	19.314	<0.001	3.043	1.852	4.998
Smokers/Users in the household (Yes)	0.775	9.767	0.002	2.170	1.335	3.527
Socialize with smokers/users at the faculty (Yes)	1.752	12.415	<0.001	5.767	2.176	15.284
Constant	−4.012	51.120	<0.001	0.018		
Hosmer and Lemeshow Test	0.860					
Correct global classification	72.2%					
R^2^_Nagelkerke	0.264					

OR: Odds Ratio; CI: Confidence Interval; Sig.: significance; reference categories: use—non-user; women; year—first and second year; “Do any of the people you live with during school term smoke/use tobacco or nicotine products?”—no; “Does any member of your household smoke/use tobacco or nicotine products?”—no; “Inside your faculty, do you hang out with people who smoke/use tobacco or nicotine products?”—no. Only results with significance are shown.

**Table 5:** Intention to stop smoking.

Intention to Stop Smoking	Frequency	Percentage (%)
“I’m not thinking about quitting smoking.”	26	18.4
“I’m thinking about quitting smoking next month.”	18	12.8
“I’m thinking about quitting smoking in the next 6 months.”	19	13.5
“I’m thinking about quitting smoking in the future, but not in the next 6 months.”	78	55.3
Total	141	100

**Table 6:** Opinions and beliefs regarding e-cigarettes.

Issues Addressed	Overall Agreement % (*n*)	Agreement (*n* = 29) % (*n*)		Sig.	OR	95% CI	
		Non-User	User			Min.	Max.
“E-cigarettes are less harmful to the health of those who use them than traditional cigarettes”	27.4 (124)	24.1 (27)	44.8 (13)	**0.027**	2.56	1.09	5.99
“E-cigarettes are harmful to the health of non-users through second-hand smoke when used in enclosed spaces.”	52.4 (237)	39.3 (44)	34.5 (10)	0.675	0.81	0.35	1.91
“E-cigarettes are an effective method for quitting smoking”	14.2 (64)	17.9 (20)	27.6 (8)	0.242	1.75	0.68	4.52
“E-cigarettes are addictive”	69.5 (314)	67.0 (75)	69.0 (20)	0.838	1.10	0.455	2642
“E-cigarettes should be banned in enclosed spaces”	67.0 (303)	56.3 (63)	65.5 (19)	0.367	1.48	0.63	3.46
“E-cigarettes promote an idea of integration and social acceptance, or even superiority, of their users.”	44.2 (200)	38.4 (43)	37.9 (11)	0.964	0.98	0.42	2.27
“Advertising/marketing increases the number of e-cigarette users.”	56.6 (256)	58.0 (65)	44.8 (13)	0.202	0.59	0.26	1.34

OR: Odds Ratio; CI: Confidence Interval; Sig.: significance. Bold: significant results.

**Table 7:** Opinions and beliefs regarding heated tobacco.

Issues Addressed	Overall Agreement % (*n*)	Agreement (*n* = 58) % (*n*)		Sig.	OR	95% CI	
		Non-User	User			Min.	Max.
“Heated tobacco is less harmful to the health of those who use it than traditional cigarettes.”	23.2 (105)	25.3 (21)	60.3 (35)	**<0.001**	4.49	2.18	9.25
“Heated tobacco is harmful to the health of non-users due to aerosol exposure when used in enclosed spaces.”	60.4 (273)	51.8 (43)	41.4 (24)	0.222	0.66	0.33	1.29
“Heated tobacco is an effective method of quitting smoking.”	10.0 (45)	10.8 (9)	19.0 (11)	0.174	1.92	0.74	5.00
“Heated tobacco is addictive”	73.7 (333)	75.9 (63)	79.3 (46)	0.635	1.13	0.68	1.85
“Heated tobacco should be banned in enclosed spaces.”	64.6 (292)	56.6 (47)	39.7 (23)	**0.047**	0.50	0.25	1.00
“The use of heated tobacco promotes an idea of integration and social acceptance, or even superiority, of its users.”	41.4 (187)	42.2 (35)	31.0 (18)	0.179	0.61	0.30	1.25
“Advertising/marketing increases the number of heated tobacco users.”	53.8 (243)	61.4 (51)	60.3 (35)	0.895	0.96	0.48	1.90

OR: Odds Ratio; CI: Confidence Interval; Sig.: significance; Min.: minimum; Max.: maximum. Bold: significant results.

**Table 8:** Opinions and beliefs regarding the water pipe.

Issues Addressed	Overall Agreement % (*n*)	Agreement (*n* = 21) % (*n*)		Sig.	OR	95% CI	
		Non-User	User			Min.	Max.
“The water pipe is less harmful than traditional cigarettes.”	39.2 (177)	36.7 (44)	38.1 (8)	0.900	1.06	0.41	2.76
“The water Pipe is harmful to the health of non-users when used in enclosed spaces.”	40.9 (185)	27.5 (33)	33.3 (7)	0.584	1.32	049	3.55
“The water pipe helps in smoking cessation”	7.7 (35)	10.0 (12)	14.3 (3)	0.557	1.50	0.39	5.84
“The water pipe is addictive.”	32.1 (145)	25.0 (30)	23.8 (5)	0.907	0.94	0.32	2.78
“The water Pipe promotes social integration and social acceptance, or even superiority, of its users.”	36.1 (163)	25.0 (30)	19.0 (4)	0.556	0.71	0.22	2.26
“The water pipe is used in association with alcoholic beverages and illicit substances.”	36.3 (164)	35.8 (43)	47.6 (10)	0.304	1.63	0.64	4.14
“The water pipe is becoming increasingly popular among young people.”	63.1 (285)	51.7 (62)	61.9 (13)	0.386	1.52	0.59	3.93

OR: Odds Ratio; CI: Confidence Interval; Sig.: significance.

## Data Availability

The dataset used to conduct the analyses is available from the corresponding author upon reasonable request.

## Appendix A

**Table A1:** Selected questions analyzed in the present study.

	Question	Question Type
Section I		
2	Age	Single-Select Multiple-Choice Question
3	Sex	Single-Select Multiple-Choice Question
4	Faculty	Single-Select Multiple-Choice Question
5	Course	Single-Select Multiple-Choice Question
6	Year	Single-Select Multiple-Choice Question
10	“Do any of the people you live with during school term smoke/use tobacco or nicotine products?” (Consider traditional cigarettes or similar, heated tobacco, electronic cigarette, and water pipe) Answer only if you answered “Yes” to the question: “Did you change residence when you entered higher education?”	Yes/No Question
11	“Does any member of your household smoke/use tobacco or nicotine products?” (Consider traditional cigarettes or similar, heated tobacco, electronic cigarette, or water pipe)	Yes/No Question
12	“Inside your faculty, do you hang out with people who smoke/use tobacco or nicotine products?” (Consider traditional cigarettes or similar, heated tobacco, electronic cigarette, or water pipe)	Yes/No Question
14	“Have you ever smoked/used a traditional cigarette (or other tobacco or nicotine products) in your lifetime?” (Consider traditional cigarettes or similar, heated tobacco, electronic cigarette, or water pipe)	Yes/No Question
15	“How often do you usually smoke/ use tobacco or nicotine products?”	Single-Select Multiple-Choice Question
Section II		
16	Which of the following statements applies to you: “I smoked/used regularly but quit LESS than a year ago.” “I smoked/used regularly but quit, MORE than a year ago.” “I did not quit smoking/using (I smoke/use regularly).”	Single-Select Multiple-Choice Question
17	“At what age did you smoke/use for the first time?”	Open answer
19	“Which of the following have you experimented with at least once?” (Illustrative images)	Multi-Select Multiple-Choice Question
20	“Which of the following do you smoke/use REGULARLY?”	Multi-Select Multiple-Choice Question
23	“Do you think the pandemic/confinement influenced your smoking/using?”	Single-Select Multiple-Choice Question
24	“Which best describes your intentions to quit smoking/using?”	Single-Select Multiple-Choice Question
Section III	- Fagerström test	
Section IV		
32	“Have you ever used or do you use e-cigarettes?”	Yes/No Question
33	“How often do you use e-cigarettes?”	Single-Select Multiple-Choice Question
34	“Have you ever used or do you use heated tobacco?”	Yes/No Question
35	“How often do you use heated tobacco?”	Single-Select “Multiple-Choice Question
36	“Have you ever used or do you use a water pipe?”	Yes/No Question
37	“How often do you use a water pipe?”	Single-Select Multiple-Choice Question
Section V		
38	“E-cigarettes are less harmful to the health of those who use them than traditional cigarettes”	5-Point Likert Scale
39	“E-cigarettes are harmful to the health of non-users through second-hand smoke when used in enclosed spaces.”	5-Point Likert Scale
40	“E-cigarettes are an effective method for quitting smoking.”	5-Point Likert Scale
41	“E-cigarettes are addictive.”	5-Point Likert Scale
42	“E-cigarettes should be banned in enclosed spaces”	5-Point Likert Scale
43	“E-cigarettes promote an idea of integration and social acceptance, or even superiority, of their users.”	5-Point Likert Scale
44	“Advertising/marketing increases the number of e-cigarette users.”	5-Point Likert Scale
45	“Heated tobacco is less harmful to the health of those who use it than traditional cigarettes.”	5-Point Likert Scale
46	“Heated tobacco is harmful to the health of non-users due to aerosol exposure when used in enclosed spaces.”	5-Point Likert Scale
47	“Heated tobacco is an effective method of quitting smoking.”	5-Point Likert Scale
48	“Heated tobacco is addictive.”	5-Point Likert Scale
49	“Heated tobacco should be banned in enclosed spaces.”	5-Point Likert Scale
50	“The use of heated tobacco promotes an idea of integration and social acceptance, or even superiority, of its users.”	5-Point Likert Scale
51	“Advertising/marketing increases the number of heated tobacco users.”	5-Point Likert Scale
52	“The water pipe is less harmful than conventional cigarettes.”	5-Point Likert Scale
53	“The water pipe is harmful to the health of non-users when used in enclosed spaces.”	5-Point Likert Scale
54	“The water pipe helps in smoking cessation.”	5-Point Likert Scale
55	“The water pipe is addictive.”	5-Point Likert Scale
56	“The water pipe promotes social integration and social acceptance, or even superiority, of its users.”	5-Point Likert Scale
57	“The water pipe is used in association with alcoholic beverages and illicit substances.”	5-Point Likert Scale
58	“The water pipe is becoming increasingly popular among young people.”	5-Point Likert Scale
Section VI		
59	“The law banning smoking indoors is enforced at my faculty.”	5-Point Likert Scale
60	“The law banning smoking indoors is enforced in hospitals/health care facilities.”	5-Point Likert Scale
61	“Smoking should be prohibited in the entire university (including stairs, inside the college fences, even in outdoor places...).”	5-Point Likert Scale
62	“Banning smoking in enclosed public spaces, such as cafes, contributes to a decrease in the number of smokers.”	5-Point Likert Scale
